# The effects of ergonomic intervention on the musculoskeletal complaints and fatigue experienced by workers in the traditional metal casting industry

**DOI:** 10.1016/j.heliyon.2021.e06171

**Published:** 2021-02-02

**Authors:** Wahyu Susihono, I.Putu Gede Adiatmika

**Affiliations:** a Industrial Engineering Department, Faculty of Engineering, University of Sultan Ageng Tirtayasa, Banten, Indonesia; b Faculty of Medicine, University of Udayana, Bali, Indonesia

**Keywords:** Ergonomic intervention, Musculoskeletal complaints, Fatigue

## Abstract

This research aimed to evaluate the effects of ergonomic intervention on the musculoskeletal complaints and fatigue experienced by workers of the traditional metal casting industry that manually pour molten metal into molds. The workers’ physical complaints are typically in the form of musculoskeletal complaints, which include (1) an activity aspect, (2) a physical aspect, and (3) a motivational aspect. The method used in this research was stratified random sampling. The subjects (*n* = 127) were divided into three groups, namely, the process cement department (PCD) group, the loam department (LD) group, and the black sand department (BSD) group. The evaluation was carried out using questionnaires based on musculoskeletal complaints and fatigue. Meanwhile, an assessment of musculoskeletal complaints and fatigue was conducted one month before the ergonomic intervention, and then during follow-ups at one and eight months after the ergonomic intervention. The results showed that the average reduction in musculoskeletal complaints and fatigue experienced by the workers in the LD group was lower than that of the workers in the PCD and BSD groups at one and eight months after the ergonomic intervention. The positive effects of the ergonomic intervention on musculoskeletal complaints were evident in terms of the back, waist, left and right thighs, right knee, right ankle, and left foot (*p* < 0.05). The positive effects of the ergonomic intervention on the level of activity-based fatigue were felt in the body and legs, and the feeling of wanting to lie down decreased. The motivational fatigue experienced by the workers manifested as difficulty in thinking, concentrating, and controlling behavior, while the physical fatigue experienced by the workers was in the form of headaches, back pain, excessive thirst, and feeling unwell (*p* < 0.05). It can be concluded that ergonomic intervention can reduce both musculoskeletal complaints and fatigue, especially by conducting a morning briefing, using ergonomic ladles when pouring molten metal into molds, and consuming nutritious food during break times.

## 1 Introduction

The manual activities of workers in the traditional metal casting industry will not be eliminated in spite of modern technology. This industry is considered traditional because the production process is still supported by a large number of workers (labor-intensive) and the use of semi-manual furnaces. Such traditional industries can also be said to be people industries due to the workers’ lack of job skills. However, since the resulting product quality is good, the price, work productivity level, material efficiency, and production process standardization are competitive enough in the global market. Therefore, the traditional metal casting industry continues to survive to the present day [1].

The production process of the traditional metal casting industry commonly uses cupola or coal-fired furnaces. Molds are made via a manual process, i.e., a handmade process. The designing of molds and the process of pouring molten metal into said molds are carried out in groups. These activities are commonly undertaken by a minimum of eight people per group. Work systems are often difficult to change because workers choose to maintain the traditions and working system that they were taught by their predecessors; this particularly applies to the traditional metal casting industry as it is a heritage industry [2]; therefore, the biggest challenge to implement work improvements is the readiness of the company's owner to make the necessary changes as well as the workers' resistance to implement such changes [3]. Through intervention, it is necessary to consider humans as the main factor driving work improvements while still upholding the values of the work culture that have been long maintained by the workers.

A preliminary study showed that most workers who have been working for 4.5 years or who have reached the age of 30 have complaints about their health and a decreased physical condition due to the workload and the mechanism for selecting work organizations. However, these complaints are no longer felt by the workers once they have been acclimated to the daily working conditions. The primary musculoskeletal complaints felt by workers (after work) pertain to the waist, neck, and several parts of the spine (L5/S1) with secondary complaints being experienced also in the spine and neck in addition to several other body areas such as the back, shoulders, arms [4, 5], hips, knees, ankles, and lumbar [6, 7]. In some other manufacturing industries, however, the dominant complaints are in relation to the back, shoulders, hands or wrists, and knees [8]. Musculoskeletal complaints occur due to extreme physical activity, excessive use of muscle strength, load lifting that exceeds the maximum lifting capacity in an ineffective posture all day [9], high-risk work [10], the work environment [11], and manual work [12].

To date, the exact body location of each complaint has still not been identified. Complaints among workers vary from one to another, so occupational health professionals find it difficult to determine appropriate measures to prevent complaints of further work-related diseases. Musculoskeletal complaints result in lost workdays, increasing costs, work-related diseases [13], and lost work time [14]. Moreover, workers with severe musculoskeletal complaints can develop permanent disabilities that limit their chances of returning to work or of performing their daily activities in the industry [15]. As the number of musculoskeletal complaints of workers increases across several body parts, fatigue can become a serious problem, thereby leading to workers suffering from musculoskeletal complaints and fatigue every day after work.

Metal casting activities are categorized as heavy work activities. Such activities are carried out continuously every day, increasing the risk of fatigue. Although various efforts have been made to improve the work system by company management, such as implementing a shift work pattern, providing overtime pay, and offering work bonuses, a successful solution for overcoming such complaints has still not been achieved. This is because these efforts rely on business processes only, and overlook the workers’ input, thereby disallowing the workers to feel the effects. As a result, over time, their work activities return to the same conditions as before the improvement efforts. Thus, it is essential to pay attention to the needs of the users or the workers when trying to implement improvements, because a holistic participatory approach is needed to solve work system problems [16].

The various tools used in the traditional metal casting industry are traditional, with minimal technological adaptation, and in their design, more attention is focused on function than to the workers' comfort and safety. Furthermore, the design of such tools does not meet the appropriate standards, and they are not accompanied by the appropriate standard operating procedures (SOPs). Improvements in the working conditions based on business processes are not sufficient to solve the problems related to the workers’ physical complaints. Therefore, an intervention is required to ensure work organizations involve their workers and multidisciplinary experts so as to address such problems holistically.

Although there are similarities between companies in their choices of activities regarding ergonomic implementation, the effectiveness and the level of active participation during such activities differ. Moreover, the level of participation in some activities will decrease due to differences in the work culture among industries. Therefore, ergonomic intervention activities are important to establish the right activity formula.

Failure to improve working conditions occurs due to a lack of understanding, active involvement, and discipline by the participants in terms of carrying out each stage of change in the workplace. In fact, an inefficient workplace design causes exposure to physical ergonomic hazards in the workplace [17]. These constraints underlie the need for ergonomic intervention in the traditional metal casting industry, and such an intervention should involve the workers in the brainstorming and idea generation processes as well as in any decision-making related to the problem-solving proposal. A participatory approach should be used to develop an organizational intervention such that the intervention is tailored to the workers’ needs [18]. The work improvement stages use the plan–do–check–action (PDCA) concept through the systemic, holistic, interdisciplinary, and participatory (SHIP) stages. The SHIP approach has been used to improve various problems [19]. An ergonomic intervention (which reduces the occurrence of complaints about body parts [20]) aims to produce a formula for an appropriate ergonomic intervention implementation stage, especially in the traditional metal casting industry.

The workstations of the metal casting industry are generally divided into three departments, namely, the process cement department (PCD Group), the loam department (LD Group), and the black sand department (BSD Group). The distribution is adjusted to match the characteristics of the products being produced, and the differences in the product characteristics produced by each department affects the work organization design. In turn, this work organization design affects the level of musculoskeletal complaints and fatigue. Therefore, the musculoskeletal complaints and fatigue in each department need to be examined to determine the level of changes in worker performance after the implementation of an ergonomic intervention. Work performance is viewed at the level of musculoskeletal complaints in a specific anatomical area of the body and the level of fatigue in three aspects, namely, an activity aspect, a motivation aspect, and a physical aspect. The effects of ergonomic interventions are assessed based on the overall reduction in musculoskeletal complaints and fatigue.

## 2 Materials and methods

### 2.1 Research design

This research was carried out from 2017 to 2018 in the traditional metal casting industry, focusing on the activity of pouring molten metal into molds by workers. The workers usually worked without a rotation or shift pattern for 7 h per day across five working days. All employees (*n* = 331) were asked to complete a preliminary questionnaire about their musculoskeletal complaints and fatigue. An observation was made using video recordings taken from the corner of the company to monitor the workers' activities. At the end of each week, the workers were also asked to fill in a weekly record of work discomfort according to the three considered aspects. This weekly record of work discomfort was used as the basis for the workers’ consultation with the occupational health professional.

**Figure 1:**
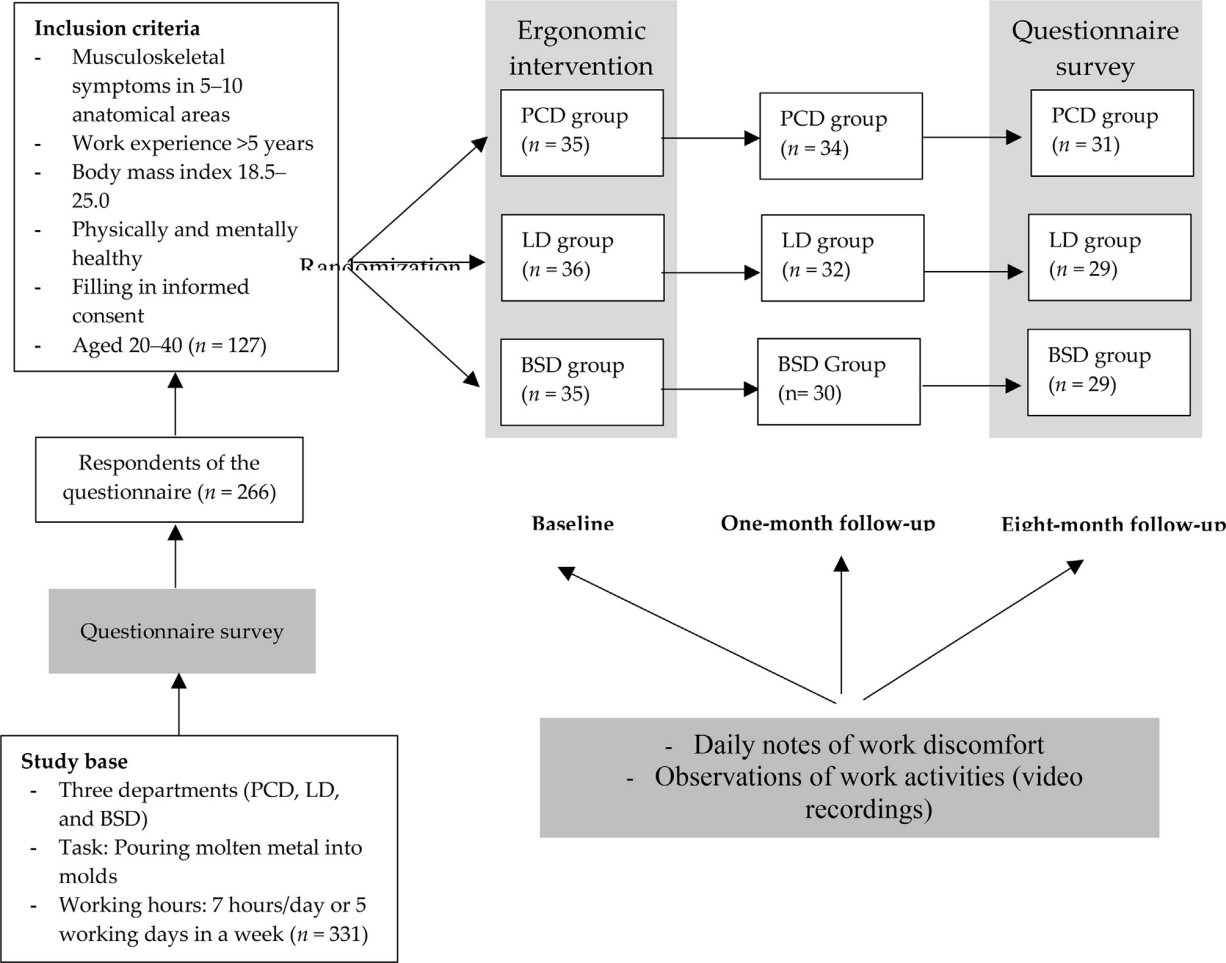
Research design. PCD, process cement department; LD, loam department; BSD, black sand department.

Subjects who met the inclusion criteria (*n* = 127) were divided into three groups, namely, the PCD, LD, and BSD groups, using stratified random sampling. Determination of inclusion criteria based on the results of periodic medical examinations by company doctors, that is symptoms of musculoskeletal complaints generally occur between the ages of 20 and 40 years, have worked for 5 consecutive years in the traditional metal casting industry. The success of the randomization was examined by considering age, weight, height, work experience, systolic and diastolic blood pressure, and body mass index (BMI). The characteristics of the groups according to data taken at the beginning of the study are presented in Table 1. The medical examination conducted by the company doctor every month. Meanwhile, the final measures of the decreasing levels of musculoskeletal complaints and fatigue were obtained from the questionnaires given to the workers at the follow-up eight months after the intervention, which are presented in Figure 1. This study was approved by the General Hospital Health Research Ethics Committee Dr. Moewardi, Faculty of Medicine-Sebelas Maret University, Number 165/II/HREC/2015 and informed consent from all participants in this study has been obtained.

**Table 1:** Characteristics of the research subject groups according to data taken at the beginning of the study.

Group	Age (years)	Weight (kg)	Height (cm)	Work Experience (year)	Systolic Blood Pressure (mmHg)	Diastolic Blood Pressure (mmHg)	Body Mass Index (kg/m^2^)
PCD group (*n* = 35)	32.7 ± 6.6	57.4 ± 3.9	162.1 ± 3.1	17.8 ± 5.2	120.7 ± 2.1	75.1 ± 3.9	21.8 ± 1.3
LD group (*n* = 36)	33.9 ± 8.0	57.9 ± 3.4	161.7 ± 3.7	16.1 ± 7.8	119.9 ± 3.3	76.9 ± 2.1	22.2 ± 1.6
BSD group (*n* = 35)	32.1 ± 6.3	58.8 ± 4.2	160.4 ± 3.3	16.6 ± 4.6	121.5 ± 3.1	74.4 ± 4.2	22.6 ± 1.5
*p*-Values	0.34	0.74	0.51	0.27	0.18	0.07	0.09

### 2.2 Ergonomic intervention

Ergonomic intervention was provided to all workers in each of the three departments (i.e., the PCD, LD, and BSD groups). In each department, the workers carried out the same activity, namely, pouring molten metal into molds. The activity started with collecting molten metal from the production kitchen, then lifting and carrying the molten metal using a 5 kg ladle to the molding station, followed by pouring the molten metal into a predetermined mold until full. The activity of pouring the molten metal was carried out, on average, eight times a day. When pouring the molten metal, the workers were continuously exposed to hot molten metal. However, the casting speed affects the quality of the molten metal mold, and errors in the workplace are fatal. Therefore, the work organization and team coordination are key to the success in terms of pouring molten metal into molds. The different activities of these three departments rely on the types of products and materials used in making the molds, and these differences affect the work organization.

The products of metal casting of the PCD group (i.e., nuts, bolts, and threads made of metal) were generally smaller in size compared to the products of the LD group. Meanwhile, the products of the BSD group (i.e., metal potted plants and metal bridgeboards) were larger in size. The difference in the size of the mold affected the work organization regarding the pouring of molten metal into the molds. Generally, the smallest-sized molds required 0.5–1.5 ladles of molten metal, the medium-sized molds required 1.5–3 ladles of molten metal, and the largest-sized molds required ≥3 ladles of molten metal. These various amounts affected the differences in the organization of teamwork, which also affected the level of musculoskeletal complaints and the fatigue experienced in the metal casting process.

The ergonomic intervention was carried out using the SHIP approach. The first step in implementing SHIP was creating a team of experts from various scientific disciplines. This team of experts discussed ergonomic problems by involving workers in order to hear their ideas. Suggestions from the workers were a determining factor in the successful identification of problems. The priority of solving the problem was determined by means of mutual agreement between a team of experts and the workers. The second step was job extension, which aimed to provide the same understanding and perception for all workers. The third step was implementing the further stages of the ergonomic intervention activities. The final step was documenting the activity in the form of an SOP. All of the intervention activities were carried out through the PDCA concept.

The form of ergonomic intervention that was mutually agreed on by the workers and the team of experts was as follows: The production leader, along with the workers in each department, conducted a morning briefing for 15–20 min. It was suggested that all workers use an ergonomic ladle with a 5 kg capacity when pouring the molten metal into molds. All workers complied with and implemented the SOPs for the tools based on a comfortable work environment.

The details of the SOPs had previously been agreed on by the workers and company management. Before carrying out any metal casting activities, the workers turned on the dust collector to reduce the impact of dust caused by this activity. The workers were advised to allow 15 min of active rest time for muscle stretching. During their active rests, they consumed food and drink (provided by the company canteen) that was recommended by the company based on nutrition calculation according to their BMI. In order to control their daily activities, every worker recorded their daily production target on the display set by the company. It was necessary for them to comply with the company recommendations regarding the use of complete personal protective equipment (PPE).

### 2.3 Subjects

A preliminary questionnaire was distributed to the workers (*n* = 331) to determine the symptoms of their musculoskeletal complaints and fatigue, and 266 of the questionnaires were returned. The common characteristics of the workers were divided into:(a) freelancer, the worker worked as a laborer only when the industry needed to conduct traditional metal casting; (b) permanent worker, who worked permanently in the traditional metal casting industry and took home a monthly salary; (c) owner and metal casting worker, who worked on a household scale.

The population of this study was selected based on the symptoms of their musculoskeletal complaints in terms of at least 5–10 anatomical areas of the body (from a total of 28 questions illustrated on the anatomical body figure) for one month prior to filling out the questionnaire. The purpose of selecting the subjects was to determine the impact of changes when carrying out the ergonomic intervention so that the benefits could be significantly felt by the workers. The subjects had to have work experience of more than five years, be physically and mentally healthy, have a BMI in the normal category, have provided informed consent, and be within the age range of 20–40 years. In this study, data on musculoskeletal complaints and fatigue were collected using a questionnaire. Furthermore, it is validated with daily notes and the results of periodic medical examinations by company doctors. However, further research using biomedical sensors such as the use of EMG will get more objective results.

Approximately 21 subjects who met the inclusion criteria refused to participate, so they were not subjected to the ergonomic intervention. At the beginning of the study, there were 106 participants, while there were only 96 participants in the one-month follow-up (10 people resigned due to long sick leave, changing jobs to other sectors, and not returning to production). Meanwhile, there were 89 participants in the eight-month follow-up (seven people resigned due to sickness, changing home address and moving to another workplace, taking long sick leave, or switching to another sector in terms of their work profession).

### 2.4 Health outcomes

The participants noted down the discomfort they felt after work, which was carried out in the afternoon. The discomfort notes were limited to complaints regarding specific anatomical parts of their bodies. These notes aimed to measure the level of consistency in any decrease in the workers’ musculoskeletal complaints, and also included any feelings of fatigue (i.e., activity, motivation, and physical fatigue) experienced by the workers. The measurement of work discomfort was carried out before the ergonomic intervention (one month prior), and then after the ergonomic intervention (follow-ups at one and eight months post-intervention). The musculoskeletal complaints were assessed using a Likert scale: 1 = not very painful, 2 = not painful, 3 = slightly painful, 4 = painful, and 5 = very painful. Meanwhile, the level of fatigue was measured using the following scale: 1 = not very tired, 2 = not tired, 3 = slightly tired, 4 = tired, and 5 = very tired. Table 4 presents a picture of the 28 anatomical areas of the body that was distributed to the research subjects with the criteria for slightly painful, painful, and very painful. Meanwhile, Table 5 presents the 30 questions to be filled by the workers (statements 1–10 were about fatigue relating to the activity aspect, statements 11–20 were about fatigue relating to the motivational aspect, and statements 21–30 were about fatigue related to the physical aspect). The highest level of fatigue in each of these three aspects was selected as a target for improvement.

## 3 Results

### 3.1 Subjects of the ergonomic intervention

Table 2 shows that the number of subjects who consistently carried out the ergonomic intervention was very varied, with the largest number of subjects in the morning briefing activity amounting to an average of 86.2% (*n* = 77); the use of ergonomic ladles when pouring molten metal into molds achieved an average of 75.2% (*n* = 67); the subjects consuming nutritious food during their active rest periods as per the company's recommended calculations based on BMI was, on average, 71.9% (*n* = 64). Changes in the number of subjects were due to several conditions experienced by the workers, namely, prolonged illness, taking leave, and stopping work.

**Table 2:** Change in the number of participants implementing activities of the ergonomic intervention (by the eight-month follow-up).

Ergonomic Intervention	PCD Group (*n* = 31) (%)	LD Group (*n* = 29) (%)	BSD Group (*n* = 29) (%)
• Conducting morning briefing	83.9	89.7	86.2
• Using ergonomic ladle (5 kg b/b capacity) when pouring molten metal into molds	80.6	82.8	62.1
• Implementing the standard operating procedures (SOPs) of the tools based on a comfortable work environment	74.2	58.6	37.9
• Implementing the SOPs of the proper production process	38.7	27.6	31.0
• Turning on the dust collector when disassembling the molds	9.7	17.2	-
• Muscle stretching during the 15 min active rest break	54.8	44.8	62.1
• Consuming nutritious food during the active rest break, according to the recommended calculations of the company based on body mass index (BMI)	71.0	75.9	69.0
• Inputting the daily production target on the display	16.1	-	72.4
• Using complete personal protective equipment (PPE)	6.5	-	20.7

During the ergonomic intervention, the activities with the lowest number of subjects was that of turning on the dust collector when disassembling a mold and using complete PPE, with an average of 9% (*n* = 8). Although the company provided the necessary equipment to facilitate these two activities, the workers’ awareness of the dangers of dust exposure were yet to become a habit in the company environment.

### 3.2 Effects of the ergonomic intervention

The musculoskeletal complaints and fatigue experienced by the workers simultaneously started to decrease, especially among the workers in the LD group. Meanwhile, the musculoskeletal complaints and fatigue of the workers in the PCD and the BSD groups did not decrease by the one-month follow-up (*p* > 0.05). However, based on the results of the musculoskeletal complaints and fatigue measurement at the eight-month follow-up, all workers in the three departments experienced significant differences (*p* < 0.05). The decrease in the rate of musculoskeletal complaints and fatigue at the eight-month follow-up was caused by the ergonomic intervention, the impact of which was very significant (Table 3).

**Table 3:** The effects of the ergonomic intervention (musculoskeletal complaints and fatigue) across the three departments at the one- and eight-month follow-ups.

Group	Before Intervention			One-Month Follow-Up				Eight-Month Follow-Up			
	*n*	Mean	SD	*n*	Mean	SD	*p-*Value	*n*	Mean	SD	*p-*Value
PCD group (*n* = 35)	35	59.82	15.821	34	57.86	9.087	0.795	31	46.71	10.801	0.000
LD group (*n* = 36)	36	50.38	9.087	32	49.64	9.531	0.000	29	43.14	7.122	0.000
BSD group (*n* = 35)	35	61.54	9.531	30	60.57	9.613	0.999	29	47.89	9.427	0.000

### 3.3 Musculoskeletal complaints

The workers across the three departments were not able to feel a significant decrease in their musculoskeletal complaints by the one-month follow-up. The decrease in the level of musculoskeletal complaints of the workers in the PCD group occurred in the lower area of the neck, while that of the workers in the LD group was in the right leg, and that of the workers in the BSD group was in the waist and right leg (*p* < 0.05). The musculoskeletal complaints had similarities with the daily log data of the workers. Complaints regarding some areas of the body in which the workers felt pain (i.e., slightly and very painful) were also written down by the workers in the daily notes. The complaints written in the daily notes were then used for comparison with the questionnaire filled out by the workers. At the eight-month follow-up, the workers recorded that they no longer experienced any musculoskeletal complaints in their daily notes (*p* < 0.05).

The different areas of musculoskeletal complaints experienced by the workers were caused by differences in the work organization of each department. Although the ergonomic intervention was the same across the three departments, the work organization and the workers’ activities in each department were different, which affected the complaints felt by each worker.

At the eight-month follow-up, different results were obtained compared to that of the one-month follow-up. The workers tended to experience a decrease in musculoskeletal complaints in some areas of the body, which occurred simultaneously across the three departments (i.e., the PCD, LD, and BSD groups). Some of the areas of the body in which the workers experienced a decrease in musculoskeletal complaints were: back, waist, left and right thighs, right ankle, and right leg (*p* < 0.05). The mean value for musculoskeletal complaints in the PCD group was 46.7 ± 8.7, in the LD group was 43.1 ± 5.7, and in the BSD group was 47.8 ± 7.4.

### 3.4 Common fatigue and fatigue in terms of the three aspects

Cumulative common fatigue was caused by three factors, namely, activity fatigue, motivational fatigue, and physical fatigue. Based on Table 4, the mean of the common fatigue experienced by the workers in the PCD group was 50.0 ± 15.8, in the LD group was 64.9 ± 17.2, and in the BSD group was 53.1 ± 14.4. There was a change in the level of common fatigue at the eight-month follow-up compared with the one-month follow-up. However, not all aspects of fatigue decreased as some workers still recorded statements of fatigue, meaning that the intervention had no effect on these workers (Table 4). The decrease in fatigue at the eight-month follow-up varied greatly across the three departments.

**Table 4:** Musculoskeletal complaints at the one- and eight-month follow-ups.

Body Area	One-Month Follow-Up						Eight-Month Follow-Up								
	PCD Group (*n* = 34) (%)		LD Group (*n* = 32) (%)		BSD Group (*n* = 34) (%)		PCD Group (*n* = 32) (%)		LD Group (*n* = 30) (%)		BSD Group (*n* = 31) (%)		Change (%)		
	*sp, p, vp*	*p-*Value	*sp, p, vp*	*p-*Value	*sp, p, vp*	*p-*Value	*sp, p, vp*	*p-*Value	*sp, p, vp*	*p-*Value	*sp, p, vp*	*p-*Value	PCD Group	LD Group	BSD Group
Pain/stiffness in the upper neck	(45,14,5)	0.555	(45,9,5)	0.064	(45,14,5)	0.070	(48,13,3)	0.056	(30,13,6)	0.042	(40,13,4)	0.091	−1	−11	−8
Pain/stiffness in the lower neck	(48,9,5)	0.057	(48,9,3)	0.133	(48,9,5)	0.065	(46,7,4)	0.099	(30,15,2)	0.015	(50,7,4)	0.098	−5	−13	−2
Pain in the left shoulder	(50,9,9)	0.126	(50,9,3)	0.066	(50,9,10)	0.093	(41,14,4)	0.099	(40,14,4)	0.099	(53,14,4)	0.043	−9	−4	2
Pain in the right shoulder	(46,11,9)	0.777	(20,11,3)	0.056	(46,11,9)	0.088	(41,16,4)	0.070	(26,6,2)	0.056	(32,16,4)	0.085	−5	0	−14
Pain in the left upper arm	(36,25,4)	0.855	(30,9,4)	0.097	(36,25,4)	0.082	(32,16,2)	0.046	(30,4,7)	0.096	(33,16,2)	0.058	−14	−2	−13
Back pain	(41,18,5)	0.057	(32,18,3)	0.073	(41,18,5)	0.075	(32,5,2)	0.015	(32,5,6)	0.032	(30,5,2)	0.047	−25	−23	−27
Pain in the right upper arm	(34,20,5)	0.099	(34,20,3)	0.076	(34,20,5)	0.099	(32,14,4)	0.044	(32,14,5)	0.034	(31,14,4)	0.099	−9	−5	−10
Lower back pain	(34,9,9)	0.065	(30,9,9)	0.059	(34,9,9)	0.088	(25,5,2)	0.050	(25,5,4)	0.045	(27,5,8)	0.007	−20	−14	−11
Buttock pain	(38,20,7)	0.053	(20,20,3)	0.238	(38,20,15)	0.038	(27,14,2)	0.081	(27,14,2)	0.181	(25,14,2)	0.081	−21	0	−31
Buttock pain	(46,18,9)	0.067	(30,9,9)	0.565	(46,18,9)	0.060	(45,20,4)	0.042	(20,20,5)	0.081	(32,20,7)	0.190	−5	−3	−15
Pain in the left elbow	(45,25,5)	0.961	(30,9,5)	0.977	(45,25,15)	0.092	(46,18,4)	0.061	(21,18,5)	0.067	(45,18,4)	0.077	−7	0	−18
Pain in the right elbow	(39,16,4)	0.964	(30,15,4)	0.279	(39,16,15)	0.097	(34,11,4)	0.179	(30,11,5)	0.096	(25,11,9)	0.065	−11	−3	−26
Pain in the left forearm	(32,18,4)	0.632	(22,10,3)	0.188	(32,18,4)	0.332	(29,13,2)	0.393	(20,13,2)	0.092	(29,13,2)	0.850	−11	−1	−11
Pain in the right forearm	(34,16,2)	0.845	(30,16,2)	0.911	(34,16,2)	0.056	(23,11,2)	0.146	(23,11,6)	0.097	(23,11,7)	0.222	−16	−8	−11
Pain in the left wrist	(15,18,4)	0.077	(39,18,2)	0.096	(39,18,15)	0.197	(36,14,4)	0.043	(36,14,2)	0.077	(36,14,4)	0.089	17	−9	−19
Pain in the right wrist	(34,14,5)	0.999	(21,10,3)	0.671	(34,14,5)	0.296	(36,9,2)	0.095	(36,9,1)	0.082	(35,9,8)	0.050	−7	12	−2
Pain in the left hand	(34,9,4)	0.498	(34,10,3)	0.766	(34,9,4)	0.092	(21,11,0)	0.098	(21,11,1)	0.099	(33,11,0)	0.076	−14	−14	−3
Pain in the right hand	(43,11,2)	0.071	(20,11,2)	0.479	(43,11,5)	0.379	(25,11,0)	0.069	(25,11,2)	0.022	(21,11,6)	0.005	−20	5	−21
Pain in the left thigh	(55,9,4)	0.059	(55,9,3)	0.099	(55,9,4)	0.066	(43,0,0)	0.043	(43,9,2)	0.009	(42,9,0)	0.045	−16	−14	−17
Pain in the right thigh	(45,16,4)	0.154	(45,16,2)	0.068	(45,16,4)	0.099	(34,11,2)	0.001	(34,11,3)	0.022	(33,11,7)	0.025	−18	−15	−14
Pain in the left knee	(32,11,5)	0.099	(32,11,2)	0.977	(32,11,7)	0.099	(38,5,0)	0.099	(38,5,1)	0.045	(38,6,5)	0.019	−5	−2	−1
Pain in the right knee	(41,11,5)	0.087	(41,11,5)	0.099	(41,11,5)	0.089	(34,5,2)	0.008	(34,5,2)	0.017	(34,7,2)	0.049	−16	−16	−14
Pain in the left calf	(34,14,5)	0.459	(34,14,5)	0.779	(34,14,9)	0.399	(23,16,2)	0.257	(23,16,6)	0.029	(23,12,6)	0.022	−13	−8	−16
Pain in the right calf	(36,13,2)	0.099	(36,10,2)	0.089	(36,13,2)	0.199	(27,11,4)	0.099	(27,11,4)	0.389	(27,15,4)	0.499	−9	−6	−5
Pain in the left ankle	(43,13,4)	0.899	(43,13,4)	0.941	(43,13,6)	0.094	(41,9,2)	0.043	(41,9,2)	0.099	(41,10,2)	0.077	−7	−7	−9
Pain in the right ankle	(39,18,4)	0.059	(39,10,4)	0.189	(39,18,4)	0.099	(32,9,2)	0.025	(32,9,5)	0.027	(32,11,2)	0.050	−18	−7	−16
Pain in the left leg	(21,16,7)	0.326	(21,10,7)	0.043	(21,16,7)	0.055	(21,13,0)	0.066	(21,13,4)	0.053	(21,11,0)	0.035	−11	−1	−12
Pain in the right leg	(27,14,5)	0.056	(27,14,5)	0.075	(27,14,4)	0.029	(18,11,0)	0.001	(18,11,3)	0.036	(18,13,0)	0.042	−18	−15	−14

Note: *sp, p, vp* = slightly painful, painful, and very painful. PCD, process cement department; LD, loam department; BSD, black sand department.

The decrease in common fatigue experienced by the workers across the three departments was very significant (*p* < 0.05) at the eight-month follow-up. However, only a few feelings can represent the decrease in fatigue. In other words, at the eight-month follow-up, the workers across the three departments simultaneously experienced changes in the form of decreasing fatigue based on three aspects (i.e., activity, motivational, and physical fatigue). The decrease in the activity fatigue of the workers included the feeling of tiredness all over the body, heavy legs, and feeling like lying down (*p* < 0.05). Meanwhile, the decrease in the motivational fatigue of the workers included difficulty thinking, feeling nervous, feeling unable to concentrate, and feeling unable to control attitudes (*p* < 0.05). The decrease in the physical fatigue of the workers included having headaches, feeling back pain, feeling thirsty at work all the time, and feeling unwell despite being medically healthy (*p* < 0.05).

## 4 Discussion

This study established a new formula for the implementation of ergonomic intervention, especially for workers in the traditional metal casting industry. The decrease in musculoskeletal complaints and fatigue as an effect of ergonomic intervention was very significant for the workers in the LD group at the one- and eight-month follow-ups after implementation. However, at the eight-month follow-up, it was shown that all workers (i.e., including in the PCD and BSD groups) experienced a decrease in musculoskeletal complaints and fatigue. Several other studies have shown differing results based on data collection during ergonomic intervention follow-ups that were used to determine changes resulting from the effects of ergonomic intervention. Some of the effects manifest after two months following the intervention [21], while others are not felt until 4, 8, or 12 months after the intervention [22], or even after 12 months [23, 24], 18 months [25], 22 months [26], or 24 months [27] following the intervention. Changes determined based on data collected during follow-ups are influenced by differences in the characteristics of the work carried out by the research subjects.

The huge changes experienced by each worker are a result of work adaptation. Work adaptation requires different lengths of time when changing between one type of work to another, and is influenced by the age of the worker, which affects the length of time needed for changes as part of organizational adaptation. In this study, the changes in work organization through ergonomic intervention had a positive effect on employee performance in the form of decreasing musculoskeletal complaints and fatigue. There is a relationship between a participatory ergonomic intervention and a reduction in musculoskeletal complaints [28, 29]. Participatory organizational intervention in the workplace increases the team's social capital in relation to change effort, and such an intervention in the workplace is a cost-effective strategy in terms of organizational readiness for organizational changes [30].

In this study, changes in work organization in the form of an ergonomic intervention included the use of ergonomic ladles when pouring molten metal into molds, discipline and implementation of SOPs when using tools, and the use of active rest time for muscle stretching. Ergonomic ladle is a must-have tool, because it serves as the only tool for carrying molten when pouring into molds. The time used in completing the work activities of each group is different. This is influenced by the type of raw material used and the size of the mold made. The difference in product completion time has an effect on work organization. However, the application of SOPs and the stages of completion of the work process in all activity groups are the same.

This work organization aimed to change work posture and movements so as to be more effective, reduce waste in body muscle movements, and apply efficient use of muscles in certain body parts such as the head, neck, shoulders, and legs. In general, ergonomic interventions have a positive impact on improving employee working conditions. In other words, ergonomic interventions can improve employee performance [31].

The strength of this research relies on the ergonomic intervention determined by the SHIP approach, otherwise known as total ergonomics. Models of this approach have been developed and applied in several companies [32]. For example, the total ergonomics approach has been used for tool improvement [33, 34], work organization [33], and work shift arrangement [35]. This includes the total ergonomics approach for reducing fatigue as well as for increasing productivity and company profits [36].

The process of identifying problems is carried out by all parties together, and the success of the chosen solution is therefore felt by all parties. If an obstacle occurs, it can be solved together for continuous improvement. Commitment of the leader is the initial key to the success of the program [37], and it requires program development through active participatory workers [38], gradual evaluation [39], joint process redesign, training, and the re-design of work organization [40].

In this study all systems were identified. The work organization system affects work activities that cause complaints experienced by workers. A holistic approach characterized by the application of ergonomic interventions to improve work organization systems in the future [41]. This study involved experts including ergonomists, economists, material experts, environmentalists, and occupational nutritionists. All of these experts form a team in charge of formulating and evaluating musculoskeletal complaints and work fatigue and their causes. Participatory ergonomics aims at arranging and prioritizing intervention steps [42]. The key to the participatory application of ergonomics lies in the participation of all parties. All parties provide input to determine priorities for the application of ergonomic interventions. Ergonomic intervention alternatives are determined collectively and consider various benefit points of view. Ergonomic interventions are based on the needs of workers, so this participatory approach starts from asking the user directly about the alternative solutions chosen [43].

Ergonomic intervention is more effective when followed by the implementation of company policies based on employee suggestions, such as morning briefings, the use of ergonomic ladles, and the provision of nutritious food. The success and sustainability of the ergonomic intervention can be guaranteed since all ergonomic interventions are the result of brainstorming by the employees themselves. Thus, a strong desire to ensure this program's success is one of the motivations foreach worker. Herein, it was demonstrated that at the eight-month follow-up after the implementation of ergonomic intervention, there was a positive effect on the level of musculoskeletal complaints and fatigue in all workers. However, the effect of musculoskeletal complaints and fatigue on workers in the LD group was lower than that of the workers in the PCD and BSD groups.

There was a positive effect of implementing the ergonomic intervention on the conditions of the workers across the three groups. The ergonomic intervention in this study had the effect of reducing the workers’ musculoskeletal complaints with regard to their backs, waists, left and right thighs, right knees, right ankles, and left legs. Meanwhile, the effects of the work organization improvements in similar studies also showed a reduction in complaints of pain in the neck, shoulders, hands, lower back, and legs [44]; lower back, shoulders, and knees [45]. However, complaints in the neck and upper back had the highest prevalence rates, amounting to 55.8% and 89.9%, respectively [46]. The decrease in musculoskeletal complaints was due to changes in the work organization, as such changes can reduce complaints in the neck, shoulders, lower back, forearms, and knees [47].

The positive effect of implementing ergonomic intervention in this study was also felt by workers in terms of a decrease in fatigue. In terms of the activity aspect, it was felt throughout the body and legs as well as a decrease in the feeling of wanting to lie down. In terms of a decrease in fatigue pertaining to the motivational aspect, effects were shown on finding it difficult to think, being unable to concentrate, and being unable to control attitude. Lastly, the decrease in fatigue related to the physical aspect was experienced by workers in the form of less headaches, back pain, always feeling thirsty, and feeling unwell. The average age of the workers was 32.9 years; as this is not particularly young, age can affect the level of fatigue, which is marked by errors in work [48] and a continuous decrease in muscle activity [49], although there was a decrease in the fatigue in the average results of all workers. The cause of this fatigue was influenced by the oxygenation conditions of the muscles which, in turn, can affect the health and productivity of workers [50].

The strength of this study is that the three groups were comparable in terms of respondent characteristics, such as work experience (>5 years) and health condition (systolic and diastolic blood pressure), measured at the beginning of the study. The workers’ health condition included BMI, and the selected workers all fell into the normal category, which was determined at the beginning of the study. Work experience affects the speed of adaptation and the adjustment to new work organization. Meanwhile, a normal BMI indicates that there is control over the nutrition condition of each worker (Table 1).

The change in the number of participants (Table 1) was influenced by individual factors or the policy factors of the company. Generally, workers are disciplined in carrying out various activities within the company if the control and supervision are carried out strictly. By contrast, workers might not be disciplined enough to maintain the consistency in changes if the work culture is not run well. In this study, the effects of the intervention were determined simultaneously, and the individual contribution of each activity is unclear. However, the change in the number of participants in terms of consistency in carrying out the intervention up until the eight-month follow-up is an indicator that the intervention activities were useful and could be implemented in the metal casting industry (Table 2). Those ergonomic intervention activities that involved more than 70% of the participants were morning briefing activities, implementation the SOPs for the tools used based on a comfortable work environment, providing nutritious food during active rest that is adjusted according to the calculations of the company based on BMI, and the activity of inputting daily production targets on display. Meanwhile, the ergonomic intervention activities that involved the lowest level of participants were turning on the dust collector during the disassembly of molds, the use of 15 min active rest times for muscle stretching, and increasing awareness regarding use of PPE. Some workers do not wear safety shoes, safety helmets and masks. However, the results of the interview indicated that the worker felt comfortable. This is because workers have adapted until they are acclimated to working conditions. The company report states that there have been no serious injuries or accidents in the past 10 years.

### 4.1 Work discomfort based on the daily notes

Work discomfort can be determined from time spent in nonwork-related activity. Evaluation of work (and nonwork) activities can be conducted by playing back recordings of the activities each workday (video recordings). The workers often engaged in activities such as stealing time for a break, taking a break to relieve fatigue, and delaying the completion of the molding process. In general, the unskilled activity of each worker is different. The level of fatigue is influenced by activity, motivation to complete work, and the physical condition of the workers. The implementation of ergonomic intervention decreased the musculoskeletal complaints and fatigue of workers. Based on the results before the intervention was carried out and the statements of the workers, the musculoskeletal complaints commonly appeared after 3 h of work. However, after the implementation of ergonomic interventions, the musculoskeletal complaints commonly appeared after 5 h of work. Thus, the longer it took the workers to complain about musculoskeletal problems, the less fatigue they felt. Fatigue is felt simultaneously when workers begin to have musculoskeletal complaints. The occurrence of musculoskeletal complaints can be inhibited by the evaluation of work discomfort [51], work organizational interventions [52], and participatory implementation of ergonomic interventions [53].

In this study, the effects of ergonomic intervention were not felt by workers by the one-month follow-up, and were only felt by the eight-month follow-up. The simultaneous impact of implementing ergonomic interventions takes time. Workers need time to adapt to the new work organization. In this study (Table 3), it was shown that at the eight-month follow-up, the ergonomic intervention had a positive effect on reducing musculoskeletal complaints and fatigue. However, it is not known exactly when the ergonomic intervention began to take effect during the seven months between the first (at one month) and last (at eight months) follow-ups. The weakness of this study is that measuring the level of change following the ergonomic intervention was only carried out at two time points.

At the one-month follow-up, the three groups (the PCD, LD, and BSD groups) did not experience a decrease in musculoskeletal complaints (Table 4), but they did experience a decrease in fatigue (Table 5), such as feeling tired throughout the body, heavy legs, the feeling of wanting to lie down, having difficulty thinking, being unable to concentrate, being unable to control work attitude, having a headache, feeling pain in the back, and constantly feeling thirsty. In general, the highest decrease in the mean number of musculoskeletal complaints in each area of the body was experienced by the workers in the BSD group. The greatest decrease in musculoskeletal complaints felt by the workers was in the back, waist, left and right thighs, right knee, and right ankle. These decreases were due to improvements in the work activities and work organization in the form of a combination of sitting and standing time [54, 55].

**Table 5:** Fatigue at work at the one- and eight-month follow-ups.

	Statements	One-Month Follow-Up												Eight-Month Follow-Up								
		PCD Group (n = 34) (%)			LD Group (*n* = 32) (%)			BSD Group (*n* = 34) (%)			PCD Group (*n* = 32) (%)			LD Group (*n* = 30) (%)			BSD Group (*n* = 31) (%)			Change (%)		
		*fs*	*f*	*p-*Value	*fs*	*f*	*p-*Value	*fs*	*f*	*p-*Value	*fs*	*f*	*p-*Value	*fs*	*f*	*p-*Value	*fs*	*f*	*p-*Value	PCD Group	LD Group	BSD Group
Activity Fatigue	Head feels heavy	3	23	0.098	43	12	0.087	64	7	0.054	57	-	0.025	57	7	0.021	50	14	0.057	31	9	−7
	Feel tired all over the body	50	32	0.006	-	32	0.010	78	-	0.040	33	-	0.001	65	4	0.013	46	7	0.050	−49	37	−25
	Heavy legs	14	15	0.006	-	32	0.003	64	7	0.030	32	-	0.028	46	12	0.029	79	-	0.028	3	26	7
	Yawn frequently	-	31	0.084	32	33	0.042	43	7	0.039	57	-	0.088	14	-	0.030	29	2	0.596	26	−51	−19
	Mind feels chaotic	-	63	0.038	17	21	0.099	36	14	0.077	43	7	0.017	43	-	0.059	17	-	0.088	−13	5	−33
	Feel sleepy	-	49	0.081	-	22	0.048	79	-	0.066	64	4	0.079	64	7	0.066	21	4	0.098	19	49	−53
	Eyes feel heavy	-	50	0.041	50	-	0.409	57	7	0.089	11	2	0.099	64	14	0.088	64	-	0.099	−37	29	0
	Muscle stiffness	-	43	0.075	57	61	0.079	43	14	0.099	64	1	0.070	50	-	0.088	21	14	0.168	22	−68	−21
	Feel unstable when standing	36	7	0.063	-	63	0.132	64	-	0.099	12	-	0.099	57	-	0.199	43	21	0.077	−31	−6	0
	Always want to life down	-	64	0.001	-	21	0.036	64	7	0.002	46	6	0.038	64	-	0.015	43	14	0.006	−12	43	−14
Motivational Fatigue	Difficulty thinking	-	93	0.084	7	56	0.015	57	7	0.001	79	7	0.048	64	9	0.009	50	7	0.048	−7	10	−7
	Feel too tired to talk	-	21	0.068	-	26	0.138	36	21	0.056	79	14	0.099	72	6	0.032	64	-	0.019	72	52	7
	Feel nervous	14	66	0.021	22	1	0.008	71	-	0.009	68	7	0.049	79	-	0.009	39	-	0.009	−5	56	−32
	Difficulty concentrating	-	78	0.005	45	-	0.037	64	-	0.049	39	-	0.007	79	6	0.009	39	3	0.007	−39	40	−22
	Feel unable to focus on something	-	53	0.058	61	-	0.179	64		0.056	55	5	0.099	79	-	0.043	86	-	0.074	7	18	21
	Forgetful	14	77	0.089	58	13	0.099	64	7	0.099	86	7	0.039	57	-	0.001	39	5	0.074	2	−14	−27
	Lack of confidence	0	46		88	-		50	14		43	-		79	-		79	1		−3	−9	15
	Feel anxious about something	15	79	0.055	63	15	0.006	79	-	0.009	33	3	0.042	14	-	0.061	71	4	0.009	−58	−64	−3
	Feel unable to control attitude	-	43	0.043	88	-	0.030	79	-	0.022	32	14	0.050	64	-	0.026	85	5	0.006	3	−24	11
	Feel unable to handle work properly	14	17	0.034	75	-	0.067	57	-	0.056	56	1	0.008	50	-	0.079	86	-	0.039	26	−25	29
Physical Fatigue	Headaches	14	-	0.016	21	-	0.039	43	-	0.019	64	-	0.034	50	-	0.029	13	-	0.007	50	29	−30
	Feel stiffness in the shoulders	29	-	0.079	9	46	0.084	22	4	0.097	68	-	0.053	11	6	0.046	48	-	0.086	39	−38	22
	Back pain	78	-	0.028	5	42	0.037	57	-	0.002	68	-	0.029	79	-	0.049	43	11	0.014	−10	32	−3
	Pressure	88	-	0.044	43	33	0.098	29	-	0.033	10	4	0.038	71	-	0.08	43	14	0.043	−74	−4	29
	Thirsty	50	21	0.017	36	-	0.033	23	1	0.024	79	-	0.016	17	-	0.018	57	21	0.045	8	−19	55
	Hoarseness	65	-	0.093	14	-	0.099	79	-	0.043	70	9	0.079	65	8	0.038	50	14	0.036	14	59	−14
	Feel dizzy	-	22	0.084	7	32	0.056	30	3	0.066	17	-	0.058	57	-	0.069	29	21	0.088	−5	18	17
	Heavy eyelids	-	54	0.055	-	51	0.053	57	-	0.864	46	-	0.054	64	-	0.077	57	-	0.055	−8	13	0
	Body tremors	74	-	0.079	36	62	0.056	50	-	0.044	50	7	0.042	44	-	0.089	50	-	0.081	−17	−54	0
	Feel unwell	68	-	0.016	-	46	0.043	43	-	0.029	62	2	0.012	36	-	0.028	57	-	0.001	−4	−10	14

Note: *fs, f =* Feel slightly and feel.

In this research, it was found that seven areas of the body experienced a significant reduction in musculoskeletal complaints (*p* < 0.05) by the eight-month follow-up measure after ergonomic intervention. However, no area of the body experienced a decrease in terms of musculoskeletal complaints by the one-month (first) follow-up as measured after the implementation of ergonomic intervention. Fatigue levels did not change by the one-month (first) follow-up, but did by the eight-month follow-up. The decrease in fatigue was generally lower for workers in the LD group. The greatest decrease in fatigue felt by workers was in the motivational aspect rather than that in the physical and activity aspects. Therefore, ergonomic interventions can improve employee performance, especially with respect to the motivational aspect, which played the biggest role due to the involvement of workers within the team from the beginning of the selection of alternative intervention activities. The workers’ ideas and suggestions were considered and became the main input in every ergonomic intervention selection design. Therefore, the initial participation from each worker was the key in the successful implementation of ergonomic intervention [56].

## 5 Conclusions

The success of the ergonomic intervention in this study was determined using the SHIP approach. The result of implementing said ergonomic intervention was a reduction in complaints regarding the workers' physical condition, including musculoskeletal complaints and fatigue. Ergonomic interventions are more effective when accompanied by the implementation of company policy; therefore, in this work, the company policy was created based on the employees’ suggestions (i.e., morning briefing, the use of ergonomic ladles, and nutritious food supply). The resulting product characteristics affected the work organization. The design of the work organization had a large effect on the decrease in musculoskeletal complaints and fatigue. The musculoskeletal complaints and fatigue of the workers in the LD group were lower than those of the workers in the PCD and BSD groups at the one- and eight-month follow-ups following the ergonomic intervention. This difference was caused by differences in the characteristics of the work activities of each department, and this affected the work organization run by each group of workers. There were positive effects of the ergonomic intervention on the musculoskeletal complaints and fatigue levels of the works, with the dominant effects on fatigue being found to relate to the motivation aspect.

## Declarations

### Author contribution statement

Wahyu Susihono: Conceived and designed the experiments; Performed the experiments; Analyzed and interpreted the data; Contributed reagents, materials, analysis tools or data; Wrote the paper.

I Putu Gede Adiatmika: Analyzed and interpreted the data; Contributed reagents, materials, analysis tools or data; Wrote the paper.

### Funding statement

This research did not receive any specific grant from funding agencies in the public, commercial, or not-for-profit sectors.

### Data availability statement

Data included in article/supp. material/referenced in article.

### Declaration of interests statement

The authors declare no conflict of interest.

### Additional information

No additional information is available for this paper.
